# *Entamoeba histolytica*: Adhesins and Lectins in the Trophozoite Surface

**DOI:** 10.3390/molecules20022802

**Published:** 2015-02-09

**Authors:** Magdalena Aguirre García, Laila Gutiérrez-Kobeh, Rosario López Vancell

**Affiliations:** Departamento de Medicina Experimental, Facultad de Medicina, Universidad Nacional Autónoma de México, Dr. Balmis #148, Col. Doctores, C.P. 06726 Mexico, D.F., Mexico E-Mails: maguirregar@yahoo.com.mx (M.A.G.); lgutierr@unam.mx (L.G.-K.)

**Keywords:** *Entamoeba histolytica*, lectins, adhesins, cytotoxicity

## Abstract

*Entamoeba histolytica* is the causative agent of amebiasis in humans and is responsible for 100,000 deaths annually, making it the third leading cause of death due to a protozoan parasite. Pathogenesis appears to result from the potent cytotoxic activity of the parasite, which kills host cells within minutes. Although the mechanism is unknown, it is well established to be contact-dependent. The life cycle of the parasite alternates with two forms: the resistant cyst and the invasive trophozoite. The adhesive interactions between the parasite and surface glycoconjugates of host cells, as well as those lining the epithelia, are determinants for invasion of human tissues, for its cytotoxic activity, and finally for the outcome of the disease. In this review we present an overview of the information available on the amebic lectins and adhesins that are responsible of those adhesive interactions and we also refer to their effect on the host immune response. Finally, we present some concluding remarks and perspectives in the field.

## 1. Introduction

The protozoo *Entamoeba histolytica* is one of the most potent cytotoxic parasites known. Due to its ability to destroy human tissues, Schaudinn in 1903 coined the term histolytica. *E. histolytica* is the causal agent of amebiasis, which is primarily a disease of underdeveloped countries and is estimated to cause 100,000 deaths each year. According to the WHO, amebiasis is the third leading cause of death due to a parasitic disease [1], although it is noteworthy that the number of cases has decreased in the past decade. One explanation is that diagnostic methods can now discriminate between the two species *E. histolytica* and *E. dispar.*

Infection occurs when the host ingests cysts in contaminated food or water. After excysting, trophozoites are released in the small intestine, may colonize the large intestine and invade through the intestinal epithelium to cause colitis or liver abscesses. Also, trophozoites can encyst and cysts are excreted in feces, which start a new infection cycle.

The process of invasion of human tissues by *E. histolytica* is crucial for the pathogenesis of amebiasis. This process has been fully analyzed thanks to the availability of quantitative *in vitro* models that assess the interaction of axenically cultured amebas with different cell types. Most of these studies have led to the conclusion that glycoconjugate recognition by the parasite plays an essential role in its pathogenesis, as well as in invasion or encystment. Thus, *E. histolytica* along its life cycle needs to contact firstly the intestinal mucosal layer to colonize the intestine. Secondly, the encysting process seems to be “triggered” by certain carbohydrate-multivalent exogenous ligands and finally, the balance between the “attachment” to the mucosa and other host cells, determines the course of the disease. It is likely that the interactions between host cells and *E. histolytica* may explain the different outcomes associated with the infection by this parasite.

This review will focus in the adhesion molecules present in *E. histolytica* HM1:IMSS trophozoites. Since the referred adhesion molecules in this parasite have been specifically described as lectins or simply adhesins, it is important to clarify both terms: a lectin is a protein (other than a glycan-specific antibody) that specifically recognizes and binds to glycans, without causing a modification, whereas an adhesin is a protein present on the surface of bacteria, viruses, or parasites that binds to a ligand present on the surface of a host cell.

The first reference of an *E. histolytica* lectin was made by Kobiler and Mirelman in 1980 [2]. They reported the presence of a “lectin activity” in trophozoites of *E. histolytica* obtained from different strains axenically (HK-9, 200:NIH and HM1:IMSS) and monoxenically cultured (HU-1:MUSC). The initial sample consisted of a crude ameba extract obtained by sonication that was able to agglutinate human erythrocytes of different blood types. Its specificity, determined by inhibition with different chitin oligomers or monosaccharides such as galactose and *N*-acetylglucosamine, was for trimers and tetramers of N-acetylglucosamine. The active component was purified by affinity chromatography with chitin, although they did not show an electrophoretic analysis The strains with the greatest lectin activity were HK-9 and HU-1:MUSC, while HM1:IMSS showed the lowest lectin activity.

Later on, research in this area advanced significantly due to axenical cultures of *E. histolytica* trophozoites in the culture medium designed by Diamond in 1978 [3]. The strain of *E. histolytica* that has been mostly used is the HM1:IMSS, considered a virulent strain, whose genome has been fully analyzed [4]. The *E. histolytica* lectins and adhesins, theme of this review, have been studied using this strain.

## 2. The 220 kDa Lectin

In 1987, Rosales-Encina and coworkers [5] reported the isolation and purification of a protein with lectin-like properties from trophozoites of *E. histolytica*. The initial sample was an amebic homogenate purified by molecular filtration. The lectin was obtained by electroelution from 5% polyacrylamide denaturing gels and the electrophoretic analysis showed a band with a molecular weight of 220 kDa, with no proteolytic activity in gel zymograms, but able to agglutinate human erythrocytes, mainly type B. The hemagglutination was inhibited by hyaluronic acid > chitotriose > chitin > *N*-acetyl galactosamine > galactose, which showed a specificity of the lectin for (GlcNAc)_n_. The lectin competitively inhibited the adhesion of trophozoites to monolayers of MDCK cells fixed with glutaraldehyde, although the inhibition was only 50%, suggesting the involvement of other molecules. Also, it was determined that the lectin is constituted by hydrophobic amino acids and glycosylated in 9% of its weight. Using specific antibodies, the lectin was recognized in a membrane subcellular fraction, which suggested a membrane localization of the lectin with receptor function involved in cellular or extracellular matrix adherence.

Almost simultaneously to the description of the 220 kDa lectin, Meza *et al*. [6] raised poly- and monoclonal antibodies against the lectin obtained from the HM-38 strain and showed that the polyclonal antibodies cross-reacted with the lectin obtained from the HM1:IMSS, HM:38, Laredo strains, as well as from *E. invadens*. Nevertheless, the monoclonal antibody only recognized the strains HM1:IMSS and HM:38. Both poly- and monoclonal antibodies partially inhibited the adherence of trophozoites to type B human erythrocytes and to monolayers of MDCK cells, as well as erythrophagocytosis. The main contribution of this work was the immunolocalization of the 220 kDa lectin in the surface of trophozoites, with differences in the distribution pattern owed to fixation. The presence of the lectin in the amebic membrane was consistent with the location suggested by Rosales *et al*. [5] and reinforced its role in the recognition and adherence of amebic trophozoites to target cells. Later, the immunological response of mice immunized with the native 220 kDa lectin (L220), recombinant (M-11) or urea-denatured lectin (fragments with molecular weights of 100, 80 and 47 kDa) was analyzed [7]. Spleen cells from mice immunized with L220 were unable to proliferate *in vitro* when stimulated with the same protein, while the immunization with M-11 or fragments derived from L220 rendered proliferation of the cells when stimulated with L220. By western blot, the antibodies obtained from mice immunized with the different lectins were able to recognize the L220 molecule. The production of cytokines was also assessed and it was found that immunization with L220 induced a T_H_2 response with the production by spleen cells of IL-4 and IL-10, while the immunization with M-11 or fragments induced the secretion of IL-2 and IFN-γ, characteristic of T_H_1 pattern. The conclusion of this work was that different epitopes in the structure of the L220 from *E. histolytica* produce different cellular immune responses, in addition to the humoral response previously described.

## 3. The 112 kDa Adhesin

Another molecule that has been involved in the adherence of *E. histolytica* is a 112 kDa protein described by Rodríguez and Orozco [8], when they were analyzing changes in virulence by isolating a non-phagocytic clone from a virulent phagocytic strain. They obtained different clones: clone A was directly isolated from the strain HM1:IMSS, from clone A they isolated clone C_9_ by mutagenesis with ethyl metasulphonate and resistance to ementine and from C_9_ they obtained ten mutants deficient in phagocytosis and from which clones C_98_, C_919_ y C_923_ were also deficient in adhesion. Later, in 1987 Arroyo and Orozco [9] used clones A and C_9_ to produce monoclonal antibodies and determined by indirect immunofluorescence microscopy that MAb Adh-1, Adh-2 and MAb-3 antibodies reacted with the surface of trophozoites obtained from clones A and C_9_, but not with clones C_98_, C_919_ and C_923_, deficient in adhesion and phagocytosis. By western blot MAb Adh-1 and Adh-2 recognized a 112 kDa protein present in extracts of clone C_9_, but not in extracts from the clones deficient in phagocytosis and adhesion. In addition, both antibodies inhibited the adhesion, phagocytosis and cytopathic effect of C_9_ trophozoites.

In 1992 Rigothier and coworkers [10] purified an intense band of 112 kDa and two polypeptides of 50 and 70 kDa from clone A by immunoaffinity and electroelution using MAb Adh-2. The three components were recognized by the MAb Adh-2 and the treatment of the sample with diethylamine (DEA) showed that the 50 and 70 kDa-fragments came from the 112 kDa protein. Also, the 70 kDa polypeptide showed proteolytic activity.

The localization of the 112 kDa protein in the parasites during erythrophagocytosis was performed by confocal microscopy using an anti-112 kDa antibody [11], and was determined that the 112 kDa adhesin is present in vacuoles and in the plasma membrane of trophozoites. Interestingly, this localization changes when trophozoites are in contact with erythrocytes [12]. In this case the presence of the adhesin in vacuoles diminishes and concentrates focally in the sites of contact between the trophozoite and the erythrocytes, suggesting an active participation of the adhesion in erythrophagocytosis.

With the cloning of the 112 kDa-adhesin in 1999, it was possible to identify and locate the cellular adhesion domain [13]. It was also determined that the 112 kDa adhesin is formed by two polypeptides of 49 and 75 kDa encoded by different genes separated by a non-coding sequence of 188 bp. The analysis of these genes and the recombinant proteins showed that the 49 kDa polypeptide is a cysteine protease (EhCP112), the mature protease with a molecular weight of 34 kDa. Later, it was found that the recombinant EhCP112 protein (rEhCP112) digests azocasein, gelatin, type I collagen, fibronectin, and hemoglobin; undergoes self-degradation, disrupts MDCK-cell monolayers, binds erythrocytes, and is recognized by the sera of patients with amebiasis [14]. The native EhCP112 is expressed in some virulence-deficient mutants and in wild clones (clone A) is found in vesicles that are secreted in the form of the EhCPADH complex (formed by a cysteine proteinase -EhCP112- and an adhesin-EhADH112-). In addition, it also has a sequence or RGD domain (Arg-Gly-Asp), suggesting that EhCP112 binds integrins [14].

On the other hand, the 75 kDa protein (EhADH112) has a domain involved in the adherence of trophozoites to target cells. The molecular weight of the whole molecule ranges between 109 and 124 kDa, due to the posttranslational addition of carbohydrate residues to EhCP112 [13].

With the use of the recombinant protein, it was confirmed that the 112 kDa adhesin is translocated during phagocytosis from the plasma membrane to phagocytic vacuoles and, after 30 min of interaction, it returns to the plasma membrane. Due to its direct involvement in phagocytosis, it was called phagosine [13].

In the early 2000s a recombinant polypeptide, EhADH243, was identified as a molecule that may participate in adherence and virulence [15]. This polypeptide has an epitope recognized by anti-EhCPADH monoclonal antibodies, which inhibit trophozoite adherence, as well as phagocytosis and destruction of cellular monolayers. Also, it was found that it protects against experimental liver amebiasis in hamsters when the peptide was administered subcutaneously and at a dose of 120 μg.

## 4. Gal/GalNAc Lectin of *Entamoeba histolytica*

The first evidence that suggested the participation of Gal/GalNAc lectin in the adherence of *E. histolytica* trophozoites to target cells was given by Ravdin and Guerrant in 1981 [16]. They showed that pre-treatment of the parasites with *N*-acetylgalactosamine (GalNAc) or galactose (Gal) blocked both adherence and cytolisis of target cells. Later, an *E. histolytica* “*N*-acetyl-d-galactosamine-inhibitable lectin” was partially purified from the supernantant (soluble fraction) obtained by ultracentifugation of an amebic sonicate [17]. The soluble fraction was gel filtrated and the “lectin activity” of the fractions was determined through their capacity to agglutinate fixed CHO cells, as well as non-fixed erythrocytes and neutrophils. The electrophoretic analysis of the fraction with the highest activity showed four bands of molecular weights between 43 and 67 kDa. The activity of this fraction increased with the use of octylglucoside, which rendered this detergent as the best to use in the purification of the galactose/N-acetylgalactosamine-inhibitable lectin (Gal/GalNAc lectin). In 1985, Salata and Ravdin [18] determined that the soluble fraction obtained after sonicating the suspension of trophozoites had mitogenic activity on human lymphocytes. The gel filtration chromatography of the soluble fraction clearly showed that the mitogenic activity was higher in the peak of protein with the highest amebic lectin activity. The lymphocytic proliferative response produced by those active fractions was specifically inhibited by low concentrations of asialofetuin (a complex glycoprotein with three galactose terminal residues), indicating that Gal/GalNAc lectin was responsible for the mitogenic activity.

In 1986, Ravdin and collaborators [19] obtained monoclonal antibodies against *E. histolytica* trophozoites. From all the hybridoma cell lines producing antibodies, 35 were against *E. histolytica* s as determined by ELISA or immunofluorescence exposed to a partially purified preparation. Through an additional screening, eight subclones were obtained capable of binding CHO cells previously exposed to a partially purified preparation of the amebic lectin Gal/GalNAc. Of these, only four were capable of inhibiting the binding of amebic trophozoites to CHO cells. One of these monoclonal antibodies, H8-5, was used to purify the Gal/GalNAc lectin. This settled the table for the purification of the amebic Gal/GalNAc lectin and William A. Petri Jr. *et al*., in 1987 [20] reported the “Isolation of the galactose binding lectin that mediates *in vitro* adherence of *Entamoeba histolytica*”. For this purification, [^35^S] methionine-labeled trophozoites were separated from the culture medium (conditioned medium) by centrifugation and solubilized with octylglucoside. The detergent-solubilized amebas and conditioned medium were separately submitted to different affinity chromatography columns coupled to: galactose, Mab H8-5, and ASOR (the most potent inhibitor of amebic adherence), and the electrophoretic analysis showed that the eluted fraction of all three columns, treated with β-mercaptoethanol, consisted of a protein with a molecular weight of 170 kDa. In the case of the eluate of the ASOR column there were also some faint bands of 26 and 30 kDa. There were no differences between the lectin obtained from the solubilized trophozoites or the conditioned medium. By western blot the lectin was recognized by three monoclonal antibodies that inhibited the adherence of the ameba to the CHO cells [19], which also were able to bind to the surface of trophozoites, thus locating the lectin as a membrane protein.

Following its purification, a structural analysis was performed which revealed a glycosylated molecule with a molecular weight in non-reducing conditions of 260 kDa and a pI of 6.2 [21]. The treatment with β-mercaptoethanol gave rise to two subunits: one heavy (H) and one light (L) weighing 170 kDa and 35 kDa, respectively, with a molar ratio of 1:1. Later, the H subunit was sequenced which revealed that it consists of 1,291 amino acid residues, 26 corresponding to the transmembrane domain, 41 to the cytoplasmic domain, and the rest to the extracellular region, with abundant cysteine residues (cysteine rich region) [22,23]. The gene family of the heavy subunit consists of five genes with an 89%–95% homology [24]. On the other hand, the L subunit is encoded by 6–7 genes, whose products are isoforms with different posttranslational modifications with 79%–85% homology [25]. The main isoforms have molecular weights of 31 and 35 kDa with almost the same amino acid composition [26,27], although the 35 kDa one lacks the acyl-glycosyl-phosphatidylinositol end (GPI anchor) present in the 31 kDa isoform. The GPI anchor permits the association of the 35 kDa-subunit with the plasma membrane and seems to be essential for the formation of the heterodimer H-L [28]). The intermediate subunit (I), also known as the 150 kDa lectin, was first described by Cheng *et al*. in 1998 [29] and is associated with the 260 kDa heterodimer, although in a non-covalent way. It was originally identified as a “target” for monoclonal antibodies that block trophozoite adherence. Different experimental evidence, such as its presence in pure Gal/GalNAc lectin preparations, the presence of small amounts of the H subunit in samples of the subunit I, both obtained by affinity and immunoaffinity chromatographies [30], and its co-localization with the H and L subunits in the surface of trophozoites have proven that the I subunit is closely associated with the Gal/NAcGal. Two genes for this subunit have been cloned which encode two proteins that share 84% amino acid homology, lack CRD, but show similarities with the variant surface glycoproteins (VSPs) of *Giardia lamblia,* due to the presence of CXXC and CXC sequences [31]. It is important to say that from the identification of the intermediate subunit in 1998, the concept that Gal/NAcGal lectin was constituted by a heterodimeric protein of 260 kDa has been replaced by the one of a complex constituted by the light, heavy and intermediate subunits.

The role of different domains of the H subunit in amebic adherence has been analyzed with the use of monoclonal antibodies raised against various antigenically and functionally distinct epitopes on the heavy subunit [32]. The majority of these antibodies inhibited the adherence of trophozoites to human colon mucins, however; at least two of them enhanced it. This opposite effect has been attributed to conformational changes due to ligand binding to the lectin. This interpretation has been supported by studies done by Vines and coworkers in 1998 [33] in which the cytoplasmic domain of the H subunit, which shares identity regions with the cytoplasmic tail of β2 integrin domains, was overexpressed and this resulted in a 50% decrease in adherence to target cells. This effect was abrogated using a subunit with certain mutations in the conserved residues of the cytoplasmic domain, which suggests that the amebic lectin may share a similar “signal transduction inside-out” with integrins [33]. Goldston *et al*. [34] have shown that attachment to different biological relevant ligands (such as hRBC’s or collagen) may induce the enrichment of the heavy and light subunits of the Gal/GalNAc lectin in lipid rafts at the trophozoites membrane, and that phosphatidylinositol biphosphate (PIP_2_) and increased intracellular calcium levels are involved in this process; these findings clearly suggest the activation of a signaling pathway.

In addition to its role in adherence, the Gal/GalNAc lectin has been implicated in amebic cytotoxicity to different target cells. This has been demonstrated with the apposition of amebic and target cell membranes which does not lead to cytotoxicity when the amebic lectin is inhibited with galactose (Gal) or N-acetylgalactosamine (NAcGal) [35] or if the target cell lacks this type of sugar on its surface [36,37]. Also, the use of a monoclonal antibody against epitope 1 of the H subunit blocked cytotoxicity, but not adherence [38].

The neutralization by monoclonal antibodies of different epitopes in the heavy subunit allowed the determination that all of them are found in the cysteine-rich region (amino acids 482–1138). The antibodies that blocked or increased the sugar-binding capacity were mapped between residues 428–818 [39], and the carbohydrate recognition domain (CRD) was found between residues 895–998 of the170 kDa subunit [40]. The localization of the CRD in this region was suggested due to the immunoreactivity of the fragment to a monoclonal antibody inhibitor of adherence and binding of NAcGal_19_BSA in a way inhibitable by NAcGal. However, Pillai *et al*. [41] found that, although the CRD is found in the cysteine-rich region, the residues needed for a high affinity binding to the sugar are 356–480 and/or 900–1143.

On the other hand, using stable transfectants of the virulent strain in which the expression of the 35 kDa subunit was inhibited by antisense RNA it was shown that the absence of the L subunit strongly inhibited their cytopathic and cytotoxic activity as well as their ability to induce the formation of liver lesions in hamsters [42,43]. These effects are also shared with the cytoplasmic domain of the H subunit of the lectin [32]. It has been suggested that this subunit has an essential role in tissue invasion by the parasite, mainly involved in the migration of *E. histolytica* through the hepatic parenchyma [44,45].

The effects elicited by the Gal/GalNAc lectin in the adherence and citotoxicity of trophozoites are clearly a result of its surface localization. Nevertheless, several *in vitro* studies, demonstrating the transfer of the lectin from the surface of trophozoites to enterocytes and hepatocytes in culture [46] and others performed using *in vivo* models such as experimental liver abscess in hamsters or intestinal amebiasis in mice [47,48] have raised the possibility that this amebic lectin could be a soluble protein. Supporting the notion that the Gal/GalNAc could be secreted, Baxt and cols. [49] investigated the function of the rhomboid family of proteases in *E. histolytica* and found that it encodes only one rhomboid protein (termed EhROM1) with the residue requirements for proteolytic activity. EhROM1 displayed the atypical mode of substrate specificity, analogous to the rhomboid of *Plasmodium falciparum* (PfROM4), the specific substrate for EhROM1 resulted to be the Gal/GalNAc lectin, as demonstrated by cotransfection of EhROM1 and the heavy subunit of Gal/GalNAc in COS cells. In *E. histolytica* trophozoites, EhROM1 changed localization to vesicles during phagocytosis and to the posterior cap structure during surface receptor shedding, in both cases colocalizing with the Gal/GalNAc lectin. These findings implicate EhROM1 in immune evasion.

The participation of this lectin in the adherence of trophozoites to mucin oligosaccharides [50] is a relevant host defense mechanism in which the lectin is implicated. This has been recently confirmed by Kato *et al*., who showed that the presence of sialic acid in colorectal mucins correlates with the susceptibility to *E. histolytica* infection in different mouse strains [51]. Also, its role in eliciting an immune response has been thoroughly studied, some of the most relevant being summarized below. It has been shown that through its CRD, the Gal/GalNAc lectin has the capacity to induce a proinflammatory response in macrophages [52,53,54,55] and Toll-like receptor signaling in human colonic cells [56]. Also, it has been recently demonstrated that the Gal/GalNAc lectin, in a contact-dependent manner and probably through PRR’s (pattern recognition receptors), is capable of inducing the activation of the inflammasome in macrophages [57]. *In vitro*, the Gal/GalNAc lectin has been shown to induce IL-2 and IFN-ɣ in T cells, eliciting a T_H_1 response [58]. The precise region of the lectin responsible of the IL-12 induction in human macrophages has been determined [59]. Interestingly, it has also been ascribed to this lectin the ability of directly initiating maturation and activation of dendritic cells characterized by T_H_1 cytokine production [60].

Braga and coworkers showed its implication in host evasion mechanisms in 1992 [61] through its role in the evasion of amebas to human complement. Although it was accepted that sequence similarity and antigenic cross-reactivity with CD59 were responsible of this effect, later, it was found that amebas can also resist complement attack through the acquisition of complement regulatory proteins from erythrophagocytosis [62]. Recently, it has been suggested that a 21 kDa protein in the surface of trophozoites has the same function [63].

With respect to humoral immune response, it has been shown that IgG and IgA against the lectin can be detected in patients with amebiasis [64,65,66]. On the other hand, there is a great deal of evidence of sIgA recognizing Gal-lectin (or a specific domain of it) based protection against *E histolytica* infection [67,68,69,70]. This lectin is the major vaccine candidate against *E. histolytica,* and has been tested in different experimental animal models of amebiasis [71,72,73,74,75].

## 5. Conclusions and Perspectives

So far we have restricted our review to those *E*. *histolytica* molecules that have been specifically ascribed as adhesins or lectins; notwithstanding other molecules that have been implicated in the adhesion process. Although the precise mechanism of cytotoxicity has not been fully defined, it is well established that after adherence, host proteins become dephosphorylated and host intracellular calcium increases [76]. Very recently it has been reported that amebas kill by ingesting pieces of living human cells (trogocytosis), which is required for cell killing, and also contributes to invasion of intestinal tissue [77]. Whatever it is the mechanism, it is clear that the principal mediator of adherence is the amebic Gal/GalNAc lectin and that contact through it is critical for the killing activity of the parasite.

Several studies have suggested that the Gal/GalNAc lectin is capable of activating signal pathways as a result of contact, to this respect, only the group of Galván-Moroyoqui and Meza [56] clearly demonstrated that the CRD of the lectin binds to a specific receptor: TLR’s and activates through these receptors a signaling pathway in the target cell, so it seems that the CRD binds a specific ligand. One aspect that has not been investigated is the fact that the heavy subunit of the Gal/GalNAc lectin, more precisely, the CRD has sequence identity with the receptor binding domain of the hepatocyte growth factor (HGF) which sets the possibility of c-Met (the receptor for HGF) being another ligand for the lectin, once again pointing for a dual role of this lectin; above all, if we take in account the important role of HGF in liver repair and regeneration during organ injury (as in amoebic liver abscesses) and on the other hand, the marked tropism of this parasite by the liver.

Something that draws attention is the fact that there are multiple genes encoding each of the three subunits of the Gal/GalNAc lectin complex; so that multiple combinations can be made to constitute de heterodimer in association to the intermediate subunit. Slight differences in the sequences of the subunits of the lectin complex embedded at the surface of the ameba may account for differences in some functions and might be responsible for virulence or avirulence and finally define the outcome of the disease. Biochemical studies of the Gal/GalNAc lectin complex are needed to understand how it works, and could be applied to other parasites and to other pathologies.
